# Cystine/Glutamate Antiporter in Schizophrenia: From Molecular Mechanism to Novel Biomarker and Treatment

**DOI:** 10.3390/ijms22189718

**Published:** 2021-09-08

**Authors:** Chung-Chieh Hung, Chieh-Hsin Lin, Hsien-Yuan Lane

**Affiliations:** 1 Institute of Clinical Medical Science, China Medical University, Taichung 40402, Taiwan; alouette_rouge@yahoo.com.tw; 2 Department of Psychiatry, China Medical University Hospital, Taichung 40402, Taiwan; 3 Graduate Institute of Biomedical Sciences, China Medical University, Taichung 40402, Taiwan; 4 Department of Psychiatry, Kaohsiung Chang Gung Memorial Hospital, Chang Gung University College of Medicine, Kaohsiung 83301, Taiwan; 5 School of Medicine, Chang Gung University, Taoyuan 33302, Taiwan; 6 Department of Psychology, College of Medical and Health Sciences, Asia University, Taichung 41354, Taiwan

**Keywords:** cystine/glutamate antiporter, system x_c_^−^, schizophrenia, biomarker

## Abstract

Glutamate, a crucial excitatory neurotransmitter, plays a major role in the modulation of schizophrenia’s pathogenesis. New drug developments for schizophrenia have been prompted by the hypoglutamatergic hypothesis of schizophrenia. The cystine/glutamate antiporter system x_c_^−^ is related to glutamate-release regulation. Patients with schizophrenia were recently discovered to exhibit downregulation of x_c_^−^ subunits—the solute carrier (SLC) family 3 member 2 and the SLC family 7 member 11. We searched for relevant studies from 1980, when Bannai and Kitamura first identified the protein subunit system x_c_^−^ in lung fibroblasts, with the aim of compiling the biological, functional, and pharmacological characteristics of antiporter x_c_^−^, which consists of several subunits. Some of them can significantly stimulate the human brain through the glutamate pathway. Initially, extracellular cysteine activates neuronal x_c_^−^, causing glutamate efflux. Next, excitatory amino acid transporters enhance the unidirectional transportation of glutamate and sodium. These two biochemical pathways are also crucial to the production of glutathione, a protective agent for neural and glial cells and astrocytes. Investigation of the expression of system x_c_^−^ genes in the peripheral white blood cells of patients with schizophrenia can facilitate better understanding of the mental disorder and future development of novel biomarkers and treatments for schizophrenia. In addition, the findings further support the hypoglutamatergic hypothesis of schizophrenia.

## 1. Introduction

Schizophrenia is a chronic brain disease affecting approximately 1% of the world population that causes a severe health burden [1,2]. Many theories and studies have argued for the role of an imbalance in serial neurotransmitters, such as dopamine and glutamate, in the pathophysiology of schizophrenia. In all these studies, increased dopaminergic and decreased glutamatergic neurotransmissions have been hypothesized to play crucial roles in the neuropsychiatric etiology of schizophrenia [3,4]. The symptoms of schizophrenia include psychotic episodes, such as delusions and hallucinations, and cognitive impairment, leading to social withdrawal and a lack of motivation [5]. People with schizophrenia experience mental illness and disability during the course of the disease, leading to dysfunction in their daily routine and lower life expectancy. Investigation of the underlying etiology of the disease can assist in the future development of diagnostic tools and treatment options.

Evidence concerning neurodevelopmental origin as well as genetic evidence suggest concordance with regard to interference in polygenic inheritance by N-methyl-D-aspartate (NMDA) neurotransmission in schizophrenia [5]. The human brain develops during adolescence, the period in which many schizophrenia symptoms appear or worsen, rendering the patients subject to suffering from the deterioration of executive performance, cognitive function and social interaction throughout their adulthood [6]. In addition, the genetic predisposition and environmental disturbances that lead to changes or imbalances in these developmental processes may also increase the schizophrenia risk [7].

Not only hyperdopaminergic but also hypoglutamatergic hypotheses of schizophrenia pathophysiology have been studied and tested [4]. Moreover, the literature shows that patients with schizophrenia may display an abnormal mechanism in antioxidant protection in peripheral blood [8,9,10], cerebrospinal fluid (CSF) [11], and postmortem brain tissues [12,13]. Evidence also suggests that patients with schizophrenia tend to have genetic bases that result in decreased appropriate antioxidant defense systems [14]. Overall, the biomolecular pathophysiology of schizophrenia is involved in many fields, from neurotransmission to oxidative stresses.

The protein subunit system x_c_^−^, related to glutamate, is composed of a heavy-chain subunit 4F2hc, the solute carrier (SLC) family 3 member 2 (*SLC3A2*), the light-chain subunit xCT, and the SLC family 7 member 11 (*SLC7A11*) [15]. Cystine is reduced to cysteine intracellularly after its incorporation into system x_c_^−^. Cysteine is the rate-limiting substrate in the biosynthesis of the antioxidant glutathione (GSH), which is one of the major antioxidants in the brain [16]. The aforementioned pathways are involved in the cortical function through glutamatergic stimulation in the human brain. Two types of glutamate receptors are present, namely metabotropic and ionotropic receptors. The metabotropic glutamatergic receptors (mGluRs) are crucial in the initiation and modulation of glutamate neurotransmission and are composed of guanine nucleotide-binding G protein. The mGluRs are activated by glutamate to release guanosine diphosphate (GDP). Activated mGluRs can therefore influence and modulate the enzyme functions, ion channels, and vesical transports. The mGluRs are divided into eight subtypes and classified into three groups on the basis of their signaling pathways, pharmacological properties, and DNA sequences. Group 1 mGluRs increase the presynaptic glutamate release and the signaling cascade involved in phospholipase C, which cleaves phosphatidylinositol-4-5 bisphosphate into diacylglycerol and inositol 1,4,5-triphosphate, resulting in calcium release. In contrast, group 2 and group 3 mGluRs both induce the interactions with G1/o species, and the signaling proteins include adenyl cyclase, creating cyclic adenosine monophosphate (cAMP). The above mGluRs have been found at presynaptic glutamate terminals and GABA interneurons. Activation of mGluRs facilitates the potentiation of NMDA receptor (NMDAR) currents. Therefore, NMDARs are depolarized, resulting in the activation of a serine/threonine protein phosphatase that dephosphorylates mGluR and depolarizes it again [5]. Glutamate outside the cell membrane should be controlled and regulated to enhance appropriate neurotransmission. The mechanism ensures fluctuation of the altered activity of metabotropic and ionotropic glutamate receptors, resulting in cognitive processing and behavioral manifestation.

The relationship between x_c_^−^ and schizophrenia pathogenesis was investigated in a recent study [17], in which the mRNA expression of *SLC7A11* and *SLC3A2* in peripheral white blood cells was discovered to be lower in patients with schizophrenia than in healthy people. The laboratory findings were consistent with the hypothesis of hypoglutamatergic neurotransmission in schizophrenia pathophysiology. We suggest that physiological markers could play diagnostic and therapeutic roles in patients with schizophrenia.

## 2. Biomolecular Mechanisms Involved in the Hypoglutamatergic Hypothesis of Schizophrenia

### 2.1. System x_c_^−^ Protein and Its Genetic Modulation in Schizophrenia Pathophysiology

In 1980, the subunit system x_c_^−^ protein was first identified in human fetal lung fibroblasts by Bannai and Kitamura [18]. The system x_c_^−^ is a sodium-independent and chloride-dependent antiporter of the anionic forms of cystine and glutamate. It is composed of a heavy-chain subunit (4F2hc and SLC3A2) and a light-chain subunit (xCT and *SLC7A11*) [15,19]. Inside its protein structure, cystine is transformed into cysteine in a 1:1 ratio, and cysteine serves as the rate-limiting substrate for the biosynthesis of GSH, which is one of the major antioxidants in the brain [16]. Since decreased GSH was observed in older humans, GSH was postulated to play as a key role in the cognitive deficiency of aged people. [20]. The influence of the aforementioned system x_c_^−^ on the concentration inside human brain cells was hypothesized to play an important role in the modulation of many neurotransmitter pathways.

The concentration of system x_c_^−^ has been found to be higher in human brain astrocyte cells than in other brain cells [21,22,23]. The modulation of system x_c_^−^ expression has been related to many neurological and psychiatric disorders [24,25,26], including schizophrenia. Different expressions of system x_c_^−^ subunits are documented in cancer, immune responses, and neurodegenerative diseases [26]. In addition, the postmortem brain of a patient with schizophrenia showed a high system x_c_^−^ protein level in the dorsolateral prefrontal cortex compared with control brains without considerable changes in the anterior cingulate cortex and hippocampus [27]. Since system x_c_^−^ plays a critical role in glutamate release and the hypoglutamatergic hypothesis concerns the contribution to schizophrenia, it has been hypothesize that system x_c_^−^ may be involved in the pathogenesis of schizophrenia.

Recently, we investigated the mRNA expression of system x_c_^−^ obtained from peripheral blood of patients with schizophrenia. To develop convenient and efficient diagnostic tools for schizophrenia, such as peripheral gene expression as a useful surrogate for gene expression in the central nervous system (CNS), we planned to examine whether the expression of the two system x_c_^−^ subunits is altered in patients with schizophrenia. Furthermore, we wanted to know whether this expression can serve as a surrogate diagnostic biomarker. We thus measured the mRNA expression levels of *SLC3A2* and *SLC7A11* in peripheral white blood cells in well-characterized, unrelated patients with schizophrenia and healthy controls. We eliminated confounding factors caused by antipsychotic agents used in schizophrenia treatment. Altered *SLC3A2* and *SLC7A11* gene expression was found to be associated with system x_c_^−^ impairment [17]. The results showed that the two subunits in system x_c_^−^ were less abundant in patients with schizophrenia. In addition, lower activity of system x_c_^−^ was discovered to be associated with the reduction of extracellular glutamate.

The mRNA levels of the two system x_c_^−^ subunits, *SLC3A2* and *SLC7A11*, were not significantly different between drug-free patients and medicated patients. Such a limitation implies that psychotropics potentially affect the expression of these subunits, and further evaluation is required. Laboratory analysis revealed that patients with schizophrenia tend to have significantly decreased mRNA expression for the aforementioned genetic particles.

### 2.2. Relationship between GSH and System x_c_^−^

GSH is a free radical reactive oxygen species (ROS) scavenger that modulates cell metabolism. Oxidative stress causes an imbalance between ROS and antioxidant defense systems in human tissues and body fluids. Therefore, excitotoxicity resulting from oxidative stressors has been postulated to be involved in the pathogenesis of most neurodegenerative diseases. GSH is a tripeptide consisting of the amino acids glutamate, glycine, and cysteine. Inside a somatic cell, GSH plays a crucial role in antioxidation, particularly through the mechanism of cystine/glutamate antiporter system x_c_^−^ [26]. Cystine is reduced to cysteine either by intracellular GSH through the formation of a mixed disulfide intermediate or by thioredoxin reductase 1 [28]. Overall, excitatory amino acid transporters are considered to play a role in cysteine importation [29].

GSH is oxidized into GSH disulfide and then either reduced by GSH reductase in a reaction requiring the reduced nicotinamide adenine dinucleotide phosphate (NADPH) or exported from the cell by multiple-drug-resistant proteins [15]. NADPH is generated through the hexose monophosphate shunt, an alternative pathway of glucose metabolism. GSH is formed by GSH S-transferases, which are formed as secondary metabolites during cell metabolism and electrophilic xenobiotics [15,30].

### 2.3. Role of Glutamate in GSH and System x_c_^−^

Glutamate receptors are divided into two types, namely ionotropic and metabotropic receptors. The concentration of extracellular glutamate is regulated for effective neurotransmission in the brain network. Fluctuation in the extracellular glutamate concentration can lead to altered activity of metabotropic and ionotropic glutamate receptors and subsequent changes in neuronal activity, leading to the change of cognitive function and subsequent behavioral manifestations [26].

Ionotropic receptors are composed of synthetic glutamate derivatives, including NMDA and 2-amino-3-(5-methyl-3-oxo-1, 2-oxazol-4-yl) propanoic acid (AMPA). Both NMDA and AMPA receptors possess the excise channel that is stimulated for glutamate. In addition to the original glutamate binding site, NMDARs have a binding site for endogenous modulatory amino acids such as glycine and D-serine. This binding site is polyamine and redox sensitive, assisting in glutamate stimulation and neurotransmission for the integration of brain cognitive function. The neurotransmission glutamate pathway in the brain modulates its activity at multiple ionotropic and metabotropic receptors, assisting its function in human brain plasticity of ensuring learning and memory. The mechanism also influences cognitive processes and human behavior.

Extracellular glutamate concentrations are postulated to be tightly regulated to ensure adequate neurotransmission. Hence, glutamate neurotransmission represents a modulation system for controlling human neuronal plasticity, learning, and memory [31]. A literature review reported deficits in the working memory of a rodent model of schizophrenia. The above memory deficits were reversed by using the cystine prodrug N-acetylcysteine to promote the stimulation of system x_c_^−^ [27]. As serial cognitive impairments, such as memory deficits and judgmental deterioration, are one of the main features of the disease course and psychopathology of schizophrenia, the effects of N-acetylcysteine are relevant to the hypoglutamatergic hypothesis of schizophrenia.

On the other hand, in a rodent phencyclidine (PCP) model of schizophrenia, deficits in working memory were reversed by using cystine prodrug N-acetylcysteine pretreatment to enhance and facilitate the activity of system x_c_^−^ [27]. These lines of evidence implicate system x_c_^−^ in schizophrenia pathogenesis through the regulation of extracellular glutamate and GSH, which can prevent the brain from oxidative damage [31]. Hence, the function of system x_c_^−^ could be beneficial in reversing worsened cognition, deteriorated memory, and behavioral problems in patients with schizophrenia. In brief summation, system x_c_^−^ may be implicated in schizophrenia pathogenesis through the regulation of extracellular glutamate and GSH. System x_c_^−^ also plays a crucial role in the determination of the cognition and behavioral manifestations of schizophrenia. The investigation of x_c_^−^ might lead us to a better understanding of the disease nature of schizophrenia.

### 2.4. Proposed and Tentative Biomarkers for Schizophrenia Diagnosis

According to the review of the literature, decreased mRNA expression of system x_c_^−^ subunits *SLC7A11* and *SLC3A2* in patients with schizophrenia supports the hypoglutamatergic neurotransmission hypothesis regarding the pathogenesis of schizophrenia. A hypothesis of NMDA neurotransmission in schizophrenia has been proposed for some time, with a basis in evidence from and experience with the NMDAR antagonist PCP and the dissociative anesthetic ketamine, which induces psychiatric symptomatology resembling the clinical presentation of schizophrenia. Abnormal concentrations of glutamate and gamma-aminobutyric acid have been proposed to play important roles in schizophrenia. Hence, the current review indicates an effective association and provides clear evidence that system x_c_^−^ subunits *SLC7A11* and *SLC3A2* may serve as important biomarkers for schizophrenia [32,33,34].

In addition, the upstream mRNA expression of *SLC7A11* and *SLC3A2* was related to β-amyloid expression in transgenic mice and an in vitro model of human Alzheimer’s disease [35]. A rat model of Parkinson’s disease revealed a relationship with system x_c_^−^ subunits [36]. Most studies have indicated that system x_c_^−^ subunits play a crucial role in the pathophysiology of neurological and psychiatric diseases [24,37,38,39,40,41,42,43]. Psychiatric diseases, including schizophrenia, are diagnosed on the basis of the clinician’s experience and face-to-face interviews. Hence, a biomarker would be essential as an objective tool for comprehensive validation of the diagnosis of psychiatric disorders. Appropriate and effective methods could support the accuracy and efficacy of disease diagnosis and treatment. From the perspective of the hypoglutamatergic hypothesis and experimental evidence, decreased mRNA expression levels of *SLC7A11* and *SLC3A2* in peripheral blood could be developed as accurate tools for diagnosing schizophrenia.

Four variants of *SLC3A2* exist (namely transcript variants 2, 3, 5, and 6), and our previous study [17] specifically evaluated transcript variant 3 (NM_002394.5). Comprehensive and thorough studies can clarify whether all variants of *SLC3A2* are related to schizophrenia’s pathophysiology and add to the current findings. The association between the extracellular glutamate concentration and mRNA expression levels of the two system x_c_^−^ subunits was not clarified in the study. The confounding factors included the effects of additive food preservatives from daily food consumption in the Han-Chinese population. The peripheral mRNA expression levels of two system x_c_^−^ subunits, *SLC7A11* and *SLC3A2* (particularly *SLC3A2*), are decreased in patients with schizophrenia. The conclusions of this study support the findings of other studies demonstrating the hypofunction of glutamate neurotransmission in schizophrenia [5,17].

**Figure 1:**
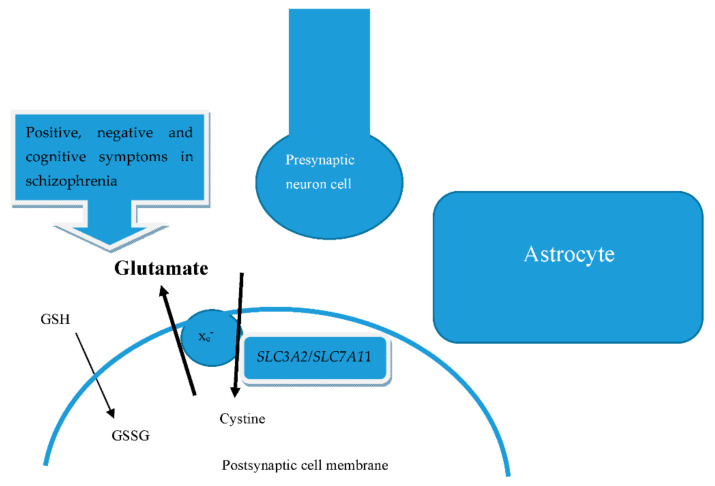
Biomolecular mechanisms between glutamate and system x_c_^−^.

We show a summary of the mentioned biomolecular in Figure 1. Glutamate and cystine are transported bidirectionally via the integral membrane protein of the antiporter system by x_c_^−^ subunits, regulated by mRNA *SLC7A11/SLC3A2*. Glutamate is hence abundant in the space formed by the presynaptic neuron cell, postsynaptic cell membrane, and astrocytes. The concentration of glutamate can therefore influence the positive, negative, and cognitive symptoms of schizophrenia. GSH also interferes with the cognitive function of schizophrenia.

## 3. Interplay between the Hypoglutamatergic Transmission and Oxidative Stress in the Etiology of Schizophrenia

NMDARs are regulated by agonists, coagonists, antagonists such as PCP and ketamine, and other molecules, including polyamines, proton, zinc, and magnesium [32]. While NMDARs can be affected by both endogenous and exogenous signals, they maintain synaptic plasticity and neuronal development in brain areas involving cognition, memory, and judgment, as well as psychosis [44,45]. NMDAR overactivation causes neurotoxicity, whereas its hypofunction results in neurodegeneration. As NMDAR hypofunction as shown to influence positive, negative, and cognitive symptoms in patients with schizophrenia, it was concluded that NMDA is an important neurotransmitter in schizophrenia [46]. Hence, NMDAR activity must be maintained within an appropriate range for human brain protection and mental health preservation [47].

Furthermore, the mechanism of increased oxidative stress contributes to the aging processes [46] and to neurodegenerative diseases through apoptotic decline in cells and tissues [12], whereas free radicals damage cells and tissues [48]. Antioxidants can assist in the prevention and reversal of cognitive deficits induced by free radicals [49]. A study has indicated cross-links among age-related NMDAR dysfunction, oxidative stress, senescence, and related forms of cognitive decline [50]. Among NMDA-related molecules, sodium benzoate works through the reduction of the activity of D-amino acid oxidase (DAO), a flavoenzyme of peroxisomes responsible for degrading D-serine and D-alanine [51,52]. Sodium benzoate can thereby inhibit ROS [53] and increase the activity of catalase, an antioxidant, in patients with schizophrenia [54]. From another perspective, pharmacological therapy with DAO results in depletion of D-serine, which attenuates NMDAR activity in cerebellar and hippocampal slices, hippocampal cell cultures, and retina preparations [55,56]. D-serine levels are decreased in the cerebrospinal fluid of drug-naive patients with schizophrenia [57]. Furthermore, both reduced brain serine racemase (SRR) and increased DAO protein levels may contribute to a decrease in CSF D-serine levels in schizophrenia [58].

In addition, a decrease in D-serine along with an increase in L-serine suggests a dysfunction in SRR activity [59] and reveals that DAO inhibitors can facilitate the effects of D-serine on prepulse inhibition (PPI) [60]. The PPI is one of the principle forms of information processing measured in schizophrenia patients and rodents treated with DAO inhibitors or NMDA antagonists. These results indicate that NMDA is associated with cognitive function in humans. Moreover, fluctuations and changes in the aforementioned SRR protein expression have been found in the postmortem brains of patients with schizophrenia [58,61,62]. Similarly, glycine levels have been found to be decreased in drug-free patients with schizophrenia and inversely correlated with the severity of negative symptoms [63]. Not only is the concentration of glycine increased but homocysteine has also been found to contribute to the pathophysiological base of schizophrenia [64]. Using high-dose glycine can damage the prepulse inhibition measure of the sensorimotor gating in humans, leading to the argument that glycine seemingly does not support cognition recovery or reservation [65].

Other regulators involved in D-serine metabolism—such as D-amino oxidase (DAO)/D-amino oxidase activator (DAOA) [58,62], the protein interacting with kinase C [66], and alanine–serine–cysteine transporter 1 [67]—are related to D-serine levels. Therefore, D-serine depletion was investigated and determined to be associated with NMDAR by its mediation of neurological functions and NMDAR-induced neurotoxicity, as well as NMDAR-dependent long-term potentiation (LTP), in many brain regions, particularly the hippocampus [57]. Brain glial cells, presumably astrocytes, pass through, with the availability of D-serine being involved in line with the deficits in synaptic learning and memory mechanisms that occur during the course of aging [68]. Overall, NMDA neurotransmission is crucial in normal human cognitive function as well as in many CNS and psychiatric disease models, forming a critical and novel hypothesis of the pathogenesis of schizophrenia.

In summary, medications acting on NMDARs can stimulate the glutamatergic reaction through various approaches and pathways in the human brain. A D-serine supplement can entirely reverse the effects of decreased NMDAR-mediated neurotransmission [55,56,68]. In human genetic studies, significant associations have been observed between the gene DAO [69] and the G72 (DAOA)/G30 [70] gene polymorphisms and schizophrenia in case–control association analyses. Additionally, the functions of these genes can be applied as therapeutic agents of schizophrenia. The therapeutic effects include not only the positive symptoms of schizophrenia but also negative symptoms or cognitive deficiencies in the course of the mental illness [71,72]. Moreover, these compounds may possess other mechanisms, such as antioxidant and mechanistic target of rapamycin (mTOR) effects, which are involved in many aspects of memory and cognition in the healthy human brain, as well as in the brains of patients with schizophrenia [71,73]. Therefore, modulators of glutamate receptors may be novel candidate targets for the treatment of refractory schizophrenia [71].

## 4. From the Invention of Biomarkers as Precision Medicine for Schizophrenia to Possible Treatment Options

The findings of the current review could lead to not only a tentative improvement in diagnostic skills but also the achievement of novel neuropharmacological therapy for schizophrenia. Neuropsychiatric drug development has opened new avenues that can help scholars understand the neurological basis of schizophrenia [7]. Although medical treatments have advanced for chronic psychiatric diseases, the diagnostic tools available remain limited. In addition, early detection and correct diagnosis of schizophrenia can decrease the disease’s severity and lead to better outcomes in patients. Advances in current neuropsychiatric pharmacology could benefit the clinical process of mental disease diagnosis and treatment outcomes. Methods for early diagnosis and improved treatment would also decrease the disease burden on societies and nations worldwide.

On the basis of the reviewed studies, we can briefly conclude that some of the candidate genes involved in the neurodevelopment and glutamate-associated signaling that are relevant to schizophrenia, such as DAO and G72 (DAOA), are directly involved in NMDA neurotransmission; the rest are indirectly related to NMDA synapses. If NMDA function can be modulated through augmentation or stimulation, poor NMDA function based on various vulnerabilities in the NMDA synapse can be reversed, regardless of its origin. Further evidence regarding the involvement of the glutamatergic system in schizophrenia would increase the understanding of the disease’s nature and open a window to a new generation of antipsychotic treatments. Considering each individual’s NMDA pathology or an accumulation of evidence on variant candidate genes, rather than any single gene, may serve as a general model for the pathogenesis of schizophrenia.

Nevertheless, on the basis of the studies reviewed herein, peripheral blood could be examined for mRNA expression of *SLC7A11* and *SLC3A2* because such expression is significantly lower in patients with schizophrenia than in healthy individuals. Furthermore, studies have suggested D-serine levels in peripheral blood as a diagnostic and therapeutic marker for schizophrenia [59]. Decreased mRNA expression of *SLC7A11* and *SLC3A2* is in accordance with the hypoglutamatergic hypothesis of schizophrenia. Hence, people with mental diseases and healthy individuals could be differentiated using well-designed biomarkers.

Our findings and review of previous studies provide new insights and perspectives for the validation of schizophrenia diagnostic tools. Newer drugs designed on the basis of the hypothesis of hypoglutamatergic and disturbed glutamate signaling in schizophrenia could contribute to crucial treatment choices in the future.

## 5. Conclusions

The genetic approaches to schizophrenia treatment are both competitive and challenging because the pathophysiology of schizophrenia is complex. Moreover, many environmental and epigenetic factors influence the nature of this disease and thus contribute to the heterogeneous manifestations of schizophrenia [44]. Regarding the hypoglutamatergic hypothesis, clinical trials have indicated that sarcosine and sodium benzoate may be more potent than glycine, D-serine, and D-cycloserine in relieving the psychopathological symptoms and signs of schizophrenia without adverse effects or safety concerns [29,54]. As the etiology of schizophrenia is multifactorial and heterogeneous [74], precise diagnosis is difficult. With the diagnostic tools for objective screening enabled by the use of the two system x_c_^−^ subunits, we might be able to improve the disease outcome of schizophrenia in the future. In addition to D-serine, D-aspartate (another endogenous NMDAR agonist enriched in the developing brain of humans) has been found to be involved in schizophrenia’s pathogenesis [75]. Reduced activity of system x_c_^−^ has been found to be linked to a reduction in extracellular glutamate levels [76]. The literature review suggests that mRNA expression of system x_c_^−^ subunits is prominent in the brains of both mice [19] and humans [77]. As oxidative stress and excitotoxicity inside cells are among the key factors associated with the neurodegenerative process, system x_c_^−^, which modulates and regulates GSH and glutamate, has been postulated to play a major role in the pathogenesis of many CNS disorders in addition to schizophrenia [78]. In transgenic mice and in vitro models of Alzheimer’s disease, the gene expression of the two system x_c_^−^ subunits *SLC7A11* and *SLC3A2* was found to be increased in the presence of β-amyloid [35]. Increased *SLC7A11* protein expression was also discovered in the striatum in a Parkinson’s disease rat model [36]. System x_c_^−^ activity was downregulated after nicotine administration in a rat model of addiction, and treatment with N-acetylcysteine to promote system x_c_^−^ activity reduced the cigarette smoking frequency in humans [79].

To briefly conclude, we propose that system x_c_^−^ subunits can serve as a diagnostic tool for schizophrenia based on the study of mRNA *SLC7A11*, and *SLC3A2*. Current studies focus on distinguishing potential biomarkers between medicated schizophrenia, drug-free schizophrenia, and healthy subjects. However, the course of schizophrenia is complicated and heterogeneous. Whether these biomarkers are significantly different in first-stage schizophrenia with refractory treatment or in other psychopathologies remains unclear. Additional studies with larger sample sizes for patients with first-episode schizophrenia and refractory treatment might be needed for clarification of the theory. Therefore, study of the mechanism through which system x_c_^−^ could serve as a diagnostic tool, as well as a therapeutic target, for schizophrenia and other CNS disorders is crucial. Research on the mRNA expression of *SLC7A11* and *SLC3A2* has revealed that diagnostic tools for schizophrenia in a clinical setting must be validated. Moreover, future development of a novel therapeutic treatment for schizophrenia, based on this mechanism, is warranted.

## Nonstandard Abbreviations

**Abbreviation **	**Full name**
AMPA	2-amino-3-(5-methyl-3-oxo-1,2-oxazol-4-yl) propanoic acid
CAT	catalase
DAO	D-amino acid oxidase
DAOA	D-amino acid oxidase activator
GDP	guanosine diphosphate
GSH	glutathione
GSSG	glutathione disulfide
LTP	long-term potentiation
mTOR	mechanistic target of rapamycin
NADPH	nicotinamide adenine dinucleotide phosphate
NMDA	N-methyl-D-aspartate
PCP	phencyclidine
PPI	prepulse inhibition
ROS	reactive oxygen species
SLC	solute carrier
SRR	serine racemase
